# A Rare Case of Radiation-Induced Osteosarcoma of the Ethmoid Sinus

**DOI:** 10.1155/2011/786202

**Published:** 2011-10-31

**Authors:** Musaed Alzahrani, Alain Robier, Yoann Pointreau, David Bakhos

**Affiliations:** ^1^Service d'ORL et Chirurgie Cervico-Faciale, CHRU Tours, 37044 Tours Cedex, France; ^2^ENT Department, King Fahad Specialist Hospital, Omar Bin Thabit St., Dammam 31444, Saudi Arabia; ^3^Université François Rabelais, 10 Boulevard Tonnellé BP 3223, 37032 Tours Cedex, France; ^4^Service d'Oncologie et Radiotherapie, CHRU Tours, 37044 Tours Cedex, France

## Abstract

Radiation therapy has been recognized as a useful modality of treatment in head and neck malignant tumors. However, radiation over 10 Gy may predispose to secondary tumors. Radiation-induced osteosarcoma of the ethmoid sinus is unusual. These tumors may present long after radiation with epistaxis. Computed tomography, magnetic resonance imaging, and biopsy are the modalities of diagnosis. We report a case of radiation-induced osteosarcoma of the ethmoid sinus 9 years after initial exposure. We describe the clinical presentation, the radiological findings, and the management.

## 1. Introduction

Ionizing radiation plays a key role in the treatment of neoplastic disease. It may, however, be followed by major side effects such as osteoradionecrosis or a second tumor. The incidence of radiation-induced sarcoma of the head and neck is likely to increase because of the progressive ageing of the population combined with the improved survival rates of head and neck cancer patients resulting from better treatment regimens [1, 2].

Osteosarcoma is a well-known, but rare complication of radiotherapy for neoplastic disease that, as a rule, occurs after doses of ionizing radiation of over 10 Gy. These tumors are uncommon but aggressive, occurring after a latency period of five years or more following radiotherapy. Histological proof of sarcoma is necessary to distinguish it from other radiotherapy changes such as osteoradionecrosis [2, 3].

We report a case of radiation-induced osteosarcoma of the ethmoid sinus, and we discuss clinical, radiological findings and the management.

## 2. Case Report

A 67-year-old man was transferred to our department for recurrent right epistaxis. His past medical history revealed an adenocarcinoma of the right ethmoid sinus 9 years prior to presentation, stage T4N0M0. He was treated by paralateronasal ethmoidectomy, followed by postoperative radiotherapy (RT) (54 Gy over 27 sessions). 

Clinical examination revealed an exophytique mass attached to the right lateral nasal wall over the sphenopalatine region. 

**Figure 1:**
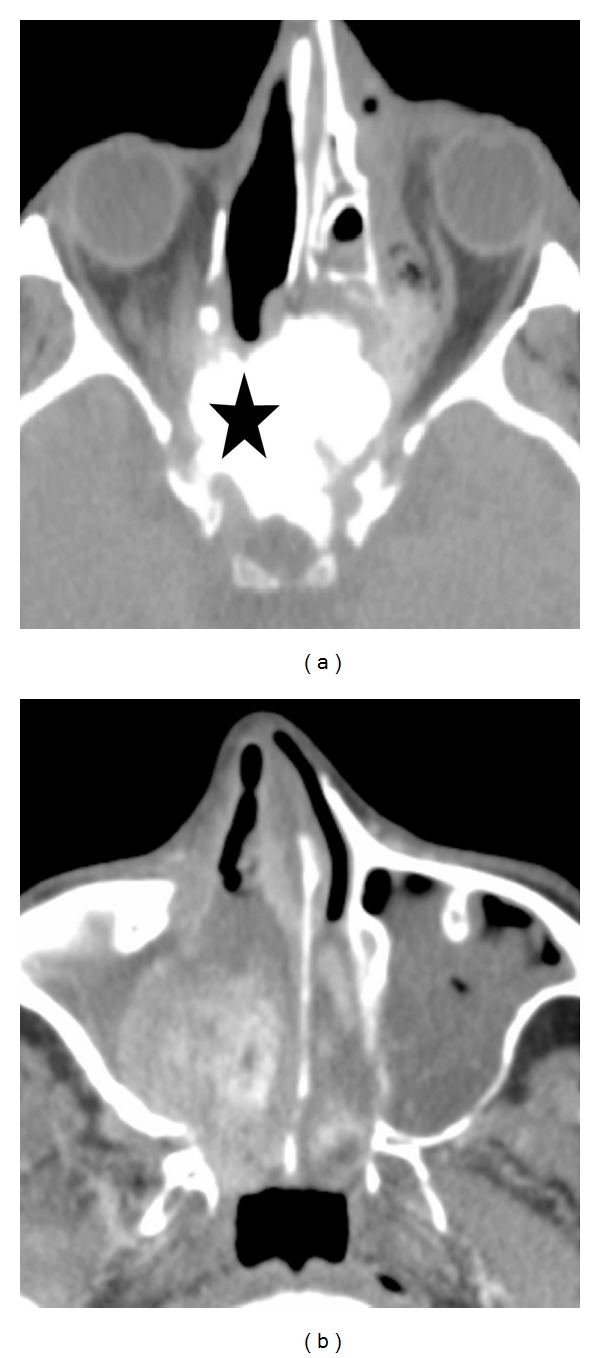
(a) Initial facial axial CT scan showing a mixed iso- and hyperdense mass (black star) of the right lateral nasal wall extending to the left orbit, (b) facial axial CT scan one month later showing progression of the tumor to involve the right maxillary sinus, the nasal fossa.

Magnetic resonance imaging (MRI) was contraindicated due to cardiac pacemaker. Computed tomodensitometry (CT) taken at admission showed a heterogeneous mass of the right lateral nasal wall extending to the left orbit with lysis of the lamina papyracea and lateral displacement of the medial rectus muscle. This mass showed mixed isodense and hyperdense signals (Figure 1(a)). 

**Figure 2:**
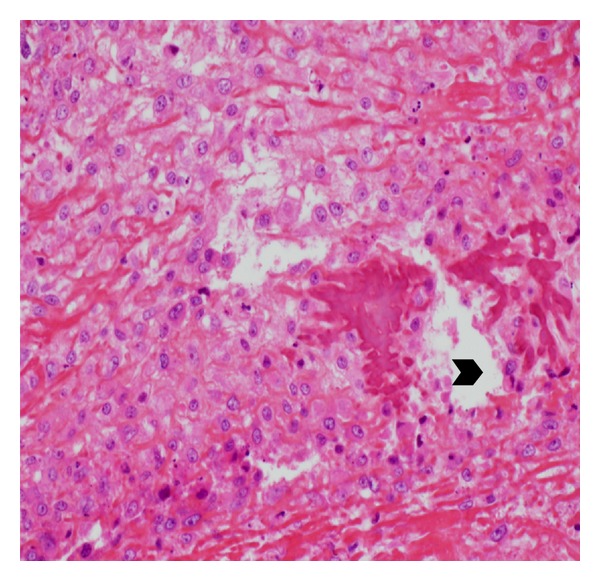
Histopathological examination showing osteoid (black arrow head) surrounded by multiple cellular atypia.

Nasal rigid endoscopy with bipolar cauterisation and excisional biopsy was realized under general anaesthesia. Histopathological examination revealed high-grade osteosarcoma (Figure 2). 

 Episodes of epistaxis continued to recur and were finally controlled by ligation of right ethmoidal and external carotid arteries. 

A further CT scan was realized one month later due to sudden loss of visual acuity of the right eye. It showed an important progression of the tumor to involve the right maxillary sinus, the nasal fossa, and the floor of the orbit (Figure 1(b)). Ophthalmology consultation concluded a tumor compression of the optic nerve in its extraconical part.

After discussing the unresectability of the tumor with the patient, chemotherapy was proposed by the multidisciplinary tumor board. However, due to altered general condition of the patient, comfort management was privileged. After a followup of 11 months, patient is still alive with stable clinical condition and no recurrence of epistaxis.

## 3. Discussion

The risk of developing an osteosarcoma after radiotherapy is rated at around 0.01–0.03% of all irradiated patients [1, 4]. Also, radiation-induced osteosarcomas account for about 5.5% of all osteosarcomas [1]. Primary osteogenic sarcomas originating from head and neck have been reported to constitute 1.6–2.7% of all cases of osteogenic sarcoma [5]. 

Most reported radiation-induced osteosarcomas of the skull base arise from the facial bone or paranasal sinuses after radiation therapy for retinoblastoma. As it is known, patients with hereditary forms of retinoblastoma may have a genetic predisposition to develop a neoplasia following radiotherapy, up to 90% in some series [3]. 

The requirements for recognition of an osteosarcoma as being radiation induced were laid down by Cahan in 1948 and later modified by Arlen in 1971: (1) the patient must have received a course of radiotherapy; (2) the tumor must arise within the area irradiated; (3) a suitably long interval must elapse between exposure to radiotherapy and tumor onset; (4) the tumor must be verified histologically; (5) there must be no preexisting disease such as neurofibromatosis, retinoblastoma, or other [6]. 

There are 3 main categories of radiation-induced tumors: low dose (<10 Gy), high dose (>30 Gy), and intermediate dose (between 10 and 30 Gy) [1].

It is commonly believed that the mean latency, that is, the interval between exposure to radiation and discovery of the tumor, is inversely proportional to the dose received. However, some authors indicate that the inverse relation between dose and latency applies only to very high doses [7, 8].

Others have noted that the interval between the course of radiotherapy and the onset of the sarcoma ranged from 3.5 to 33 years (13 years; median 10 years) [9]. In our case, it appeared 9 years after radiation.

CT scan findings include bone sclerosis and tumor calcification while MRI is useful in defining the extension into the neighbouring soft tissue. 

Histologically, this type of osteosarcoma seems to have no particular characters distinguishing it from the other types: the fibroblastic and osteoblastic varieties are the most frequent [10].

## 4. Conclusion

Radiation-induced osteosarcoma of the ethmoid sinus is potential sequelae of radiation therapy. Although rare and might be confused with recurrent disease or osteoradionecrosis, this possibility should be considered in any patient who had received radiation therapy.
